# PET Measures of D1, D2, and DAT Binding Are Associated With Heightened Tactile Responsivity in Rhesus Macaques: Implications for Sensory Processing Disorder

**DOI:** 10.3389/fnint.2019.00029

**Published:** 2019-07-17

**Authors:** Mary L. Schneider, Colleen F. Moore, Elizabeth O. Ahlers, Todd E. Barnhart, Bradley T. Christian, Onofre T. DeJesus, Jonathan W. Engle, James E. Holden, Julie A. Larson, Jeffrey M. Moirano, Dhanabalan Murali, Robert J. Nickles, Leslie M. Resch, Alexander K. Converse

**Affiliations:** ^1^Occupational Therapy Program, Department of Kinesiology, University of Wisconsin–Madison, Madison, WI, United States; ^2^Harlow Center for Biological Psychology, University of Wisconsin–Madison, Madison, WI, United States; ^3^Department of Psychology, University of Wisconsin–Madison, Madison, WI, United States; ^4^Department of Psychology, Montana State University, Bozeman, MT, United States; ^5^Waisman Center, University of Wisconsin–Madison, Madison, WI, United States; ^6^Department of Medical Physics, University of Wisconsin–Madison, Madison, WI, United States

**Keywords:** sensory processing disorder, rhesus macaque, dopamine, tactile responsivity, positron emission tomography

## Abstract

Sensory processing disorder (SPD), a developmental regulatory condition characterized by marked under- or over-responsivity to non-noxious sensory stimulation, is a common but poorly understood disorder that can profoundly affect mood, cognition, social behavior and adaptive life skills. Little is known about the etiology and neural underpinnings. Clinical research indicates that children with SPD show greater prevalence of difficulties in complex cognitive behavior including working memory, behavioral flexibility, and regulation of sensory and affective functions, which are related to prefrontal cortex (PFC), striatal, and midbrain regions. Neuroimaging may provide insight into mechanisms underlying SPD, and animal experiments provide important evidence that is not available in human studies. Rhesus monkeys (*N* = 73) were followed over a 20-year period from birth into old age. We focused on a single sensory modality, the tactile system, measured at 5–7 years, because of its critical importance for nourishment, attachment, and social reward in development. Positron emission tomography imaging was conducted at ages 12–18 years to quantify the availability of the D1 and D2 subtypes of the DA receptor (D1R and D2R), and the DA transporter (DAT). Heightened tactile responsivity was related to (a) elevated D1R in PFC overall, including lateral, ventrolateral, medial, anterior cingulate (aCg), frontopolar, and orbitofrontal (OFC) subregions, as well as nucleus accumbens (Acb), (b) reduced D2R in aCg, OFC, and substantia nigra/ventral tegmental area, and (c) elevated DAT in putamen. These findings suggest a mechanism by which DA pathways may be altered in SPD. These pathways are associated with reward processing and pain regulation, providing top-down regulation of sensory and affective processes. The balance between top-down cognitive control in the PFC-Acb pathway and bottom-up motivational function of the VTA-Acb-PFC pathway is critical for successful adaptive function. An imbalance in these two systems might explain DA-related symptoms in children with SPD, including reduced top-down regulatory function and exaggerated responsivity to stimuli. These results provide more direct evidence that SPD may involve altered DA receptor and transporter function in PFC, striatal, and midbrain regions. More work is needed to extend these results to humans.

## Introduction

The ability of the brain to receive, integrate, and respond to sensory information from an ever-changing environment is essential for adaptive behavior. Tactile defensiveness, defined as over-responsivity to tactile sensory input, was a term introduced by Jean Ayres, an occupational therapist and founder of sensory integration theory, over 50 years ago (Ayres, 1964, 1972; Ayres and Robbins, 1979). Atypical sensory integration (Ayres, 1969; Ayres and Robbins, 1979; Mailloux et al., 2011), also referred to as sensory processing disorder (SPD) (Miller et al., 2009) includes (a) over-reactivity, or heightened, aversive, or avoidant responses to sensory stimuli, (b) hypo-reactivity, or reduced, delayed or absent responses to stimuli, and (c) sensory craving, an excessive fascination or desire for sensory input [see (Williams et al., 2018)]. SPD, estimated to affect 5–16% of children (Ahn et al., 2004; Ben-Sasson et al., 2009) is associated with enduring challenges in mood, cognition, motor function, daily adaptive and social behavior, leading to impairments in family life and well-being (Bar-Shalita et al., 2008; Reynolds and Lane, 2008; Carter et al., 2011; Ben-Sasson et al., 2013; Gourley et al., 2013; Miller et al., 2017; Cascio et al., 2019). The most recent DSM-5 (American Psychiatric Association, 2013) added hyper- and hypo-sensitivity to sound and touch to the diagnostic cluster of symptoms defining autism spectrum condition (ASC). Mounting evidence indicates that SPD has overlap but is distinct from ASC (Schoen et al., 2009; Miller et al., 2012; Owen et al., 2013).

The neural mechanisms underlying atypical sensory processing function represent a fundamental unresolved question. Understanding of underlying neural dysfunction is of critical importance for effective interventions and to improve developmental outcomes for these children and their families. Some evidence indicates that children with SPD compared to typically developing children show autonomic nervous system dysregulation, observed as lower vagal tone and altered electrodermal response, and less efficient sensory gating (Mangeot et al., 2001; Kisley et al., 2004; Davies and Gavin, 2007; Schoen et al., 2009; Schaaf et al., 2010). Thus far neuroimaging studies have been limited to diffusion tensor imaging (DTI), which have implicated reduced white matter integrity in various pathways as playing key roles in SPD (Owen et al., 2013; Chang et al., 2014, 2016). For example, striking decreases were shown in posterior-located sensory projection areas that connect the higher order and multimodal sensory regions (Owen et al., 2013). In a study comparing SPD with ASD, the SPD-only group showed trends for reduced connectivity in all measured frontal tracts (Chang et al., 2014) as well as extensive white matter reductions in most of the measured tracts. Whereas ASD and SPD children showed deficient connectivity in sensory processing tracts, the impairments were more striking for the SPD group. Finally, reduced white matter correlated with parent report measures of atypical sensory behavior as well as with direct assessment of tactile and auditory processing (Chang et al., 2016).

In this paper, we present our studies on tactile responsivity and the relations of tactile responsivity to measures of the dopamine system *in vivo* in rhesus monkeys. Non-human primate models are important because they permit the advantages of randomization to experimental conditions and rigorous control over numerous environmental conditions that are often confounded in human correlational research, such as nutrition and lifestyle. Such factors can have profound effects on brain and behavioral function in humans. Non-human primates serve as excellent models for studying brain-behavior relationships because of the similarity to humans in complex cognitive and social behaviors. Also, the similarity of human and non-human primate brain structures and biological processes affords greater generalizability to human clinical conditions compared with rat studies. Primate studies fill a research gap between rodent studies and human correlational results.

We concentrated on a single sensory modality, the tactile system, because of the importance of the tactile system in primates for nourishment (rooting and sucking reflexes), contact comfort and attachment, which are considered early experiences of social reward (Harlow and Harlow, 1962; Montagu, 1984; Muir, 2002). Social touch can reduce negative affect and promote pleasurable positive feelings depending upon context and motivational state [see (Ellingsen et al., 2015)]. Evidence from human and animal studies has shown that reduced maternal and social touch causes adverse outcomes in offspring including impaired attachment and reduced cognition (Harlow and Harlow, 1962; Hertenstein et al., 2006; Wilbarger et al., 2010) for a review of classic studies of humans and animals [see (Thompson and Grusec, 1970)].

We used non-invasive *in vivo* molecular imaging by positron emission tomography (PET) to examine the dopamine (DA) system in specific brain regions in the context of two longitudinal experiments on the effects of prenatal exposure to stress and/or alcohol, compared with controls, in rhesus monkeys. We focused on the DAergic neurotransmitter system because of the importance of this system in regulating most facets of human behavior, including cognitive function, emotion regulation, motor control, reward, motivation and response to stressors. DA is one of several neurotransmitters thought to modulate social touch in mammals (Champagne et al., 2004). For example, human studies have shown that massage therapy, compared to relaxation, increases urinary measures of dopamine and serotonin (Field et al., 2005). In rats, mild non-noxious tactile stimulation in the form of stroking increased nucleus accumbens (Acb) DA signaling and effects were extinguished after lesioning the VTA (Maruyama et al., 2012). This underscores the important relation between the social touch system and the mesolimbic DA system.

Dopamine receptors are classified as D1-like receptors (D1 and D5) and D2-like receptors (D2, D3, and D4), based on their molecular structures, pharmacology, and signal transduction mechanism (Kebabian and Calne, 1979; Scheggi et al., 2018). D2R’s are found mostly in striatum, while D1R’s are widely distributed in the brain (Hall et al., 1994). D1R’s have a particularly crucial role in sustaining higher cognitive functions including attention, response inhibition, working memory, and executive function (Goldman-Rakic et al., 2004; Arnsten et al., 2015). D2Rs are involved in response to novel, salient or rewarding stimuli, response inhibition, emotion regulation, and mediation of addiction. The DA transporter (DAT) rapidly clears DA from the extracellular space, limiting the amplitude and duration of DA signaling, and maintaining homeostasis in the DA system.

In order to further understand the neural underpinnings of SPD, we tested the hypothesis that DA system function would be related to tactile processing function in rhesus monkeys. To accomplish this, rhesus monkeys from two 20-year prospective longitudinal experiments were examined using a novel behavioral assay for assessing sensory processing function in adult macaque monkeys, the Sensory Processing Scale for Monkeys (SPS-M) (Schneider et al., 2008b). We adapted procedures from sensory processing assessments for humans (Baranek and Berkson, 1994; Miller et al., 1999). In our assessment, mild repetitive tactile stimulation items were administered to the adult monkey to assess the pattern of responsivity across trials. Compared to control monkeys, the monkeys prenatally exposed to mild stress or alcohol during different gestational periods showed heightened tactile responsiveness (HTR), though the effects showed some sensitivity to gestational timing of exposures as well as serotonin transporter genotype (Schneider et al., 2008a,b).

Our series of PET studies on the animals from these two experiments were conducted to assess D1Rs, D2Rs, and DAT in the frontal-striatal circuit, an important brain region in regulatory function (Casey, 2001). We used radiotracers specific to binding to D1R, D2R, and DAT. We were particularly interested in PFC and striatum and their sub-regions because of their critical role in organizing complex cognitive function and translating stimulus properties into adaptive behavior, as well as midbrain, the location of DA cell bodies. In this paper we examined the relationships of ligand binding to our findings from the SPS-M (Schneider et al., 2008b), concentrating on brain regions that had shown effects of DA in our previous work (Schneider et al., 2008a, 2017; Converse et al., 2013).

## Materials And Methods

### Subjects

Subjects were 73 rhesus monkeys (*Macaca mulatta*) from two experiments involving prenatal stress and/or fetal alcohol exposure [see (Schneider et al., 1997, 2001) for details]. Briefly, in Expt 1, female monkey breeders were exposed to one of four prenatal treatments: (1) prenatal alcohol (voluntary daily consumption of 0.6 g/kg alcohol solution); (2) controls voluntarily consumed a solution equivolemic and equicaloric to #1; (3) mild prenatal stress (exposure to 3 loud noise bursts five times weekly; and (4) prenatal alcohol and prenatal stress (#1 plus #3). In Expt 2, female breeders were exposed to one of four prenatal treatments: (1) early gestation alcohol (daily prenatal alcohol consumption (0.6 g/kg) on gestation days 0–50); (2) mid-late gestation alcohol (gestation days 50–135); (3) continuous gestation alcohol (gestation days 0–135), or (4) control (equivolemic and equicaloric solution consumed on gestation days 0–50, 50–135 or 0–135). Infant monkeys were housed with their mothers in individual cages during the first 6 months of life. At 6 months, they were separated from their mothers for weaning and then reared in mixed-sex peer groups consisting of 5–6 monkeys from similar prenatal conditions. From 32 months of age on, the animals were pair-housed with same-sex peers. These studies were approved by and conducted in accordance with the Institutional Animal Care and Use Committee of the University of Wisconsin-Madison.

### General Procedures

All monkeys were fed a standard ration of Purina Monkey Chow (Purina Mills, St. Louis, MO, United States) supplemented three times weekly with fresh fruit. Tap water was available *ad libitum*. All animals were housed under identical conditions, undisturbed except for necessary routine animal husbandry. Lighting and temperature housing conditions were controlled with 16 h light (6 am lights on), 8 h dark, and temperature 21°C + 5°C.

### Adult Sensory Processing Scale for Monkeys (SPS-M)

The SPS-M was adapted from laboratory observational measures of sensory processing for children (Baranek and Berkson, 1994; Miller et al., 1999). The SPS-M has been described in detail previously (Schneider et al., 2008b). All animals in the study (Expts 1 and 2) underwent identical SPS-M testing, conducted when the monkeys were 5 to 7 years old. It was conducted in a 53 × 44 cm testing cage situated in a dimly lit and sound-shielded room (62 dB) with a masking white noise of 65–70 dB. Each monkey was tested individually by a human experimenter who stood beside the cage and administered a series of 18 tactile stimulation items (6 feather trials, 6 cottonball trials, and 6 brush trials, stimuli were attached to a pole) through the bars of the cage as a swipe to the cheek and neck area to assess the pattern of responsiveness across trials. Prior to the first presentation of each stimulus, the stimulus was placed in full view and touching range of the monkey and remained there for approximately 3-s. Once the animal looked at the object, the examiner slowly moved the stimulus into the cage and began the series of trials. Raters blind to the condition and history of the animals scored the subjects’ responses for degree of withdrawal from tactile stimuli in 0.25 increments on a 0 to 3 rating scale with the integers labeled as follows: 0 = no withdrawal; 1 = slight withdrawal, such as turning head away from the stimulation; 2 = moderate withdrawal, such as turning full body away from stimulation; 3 = extreme withdrawal, such as moving body away from stimulation. As described in Schneider et al. (2008b), six scores were derived that represented the mean response to the six presentations of each texture, and the linear trend of the response to each texture over the six presentations. The scores presented here are called “Sensory factor 1” in Schneider et al. (2008b). The weights in creating the factor score are 0.73 ^*^ Feather mean +0.94 ^*^ Cotton mean +0.91 ^*^ Brush mean −0.42 ^*^ Feather linear −0.27 ^*^ Cotton linear.

### Positron Emission Tomography (PET)

Positron emission tomography scans were acquired when monkeys were 12 to 18 years old as described in greater detail elsewhere (Converse et al., 2013, 2014; Moirano et al., 2018). Briefly, procedures were as follows. *Radiotracer*: D1R-type binding was measured using [^11^C]SCH 23390 (DeJesus et al., 1987), which is specific to D1. D2R-type was measured with [^18^F]fallypride (Mukherjee et al., 1995), which is specific to D2 and D3, and DAT was measured with [^18^F]FECNT (Murali et al., 2013). Monkeys were imaged in separate scans for each radiotracer. Due to multiple constraints during the two longitudinal studies, 39 of 73 subjects were imaged with all three radiotracers and assessed for tactile responsivity. Rather than discard data from subjects with incomplete measures, we analyzed data for each radiotracer from all animals that had undergone tactile assessments. *Scanning protocol*: Subjects were anesthetized with isoflurane and positioned in a microPET P4 or Focus 220 scanner with better than 2 mm full width at half maximum spatial resolution (Tai et al., 2001, 2005). Following a transmission scan, an emission scan was started and a 5 mCi bolus of radiotracer was injected intravenously. *Image reconstruction*: Emission data were temporally binned at 5 × 1, 5 × 2, and 3 × 5 min, with additional 10-min frames. The transmission scan was reconstructed to create an attenuation map. Emission images were created by filtered backprojection with corrections for detector sensitivity, dead time, radioactive decay, attenuation, and scatter. *Image processing*: Time-averaged 3D images were aligned to a labeled MRI template by affine transformations with nine degrees of freedom, equivalent to shifts, rotations, and zooms in three axes. The resulting transformations were applied to the 4D images (Jenkinson et al., 2002). Motion correction was applied as needed. Time-activity curves were determined for anatomically defined regions of interest (Moirano et al., 2018). Because of their significance in DA neural circuits, the following regions were examined: (1) PFC including subdivisions of medial PFC (mPFC), which includes anterior cingulate (aCg), lateral (lPFC), which includes ventrolateral (vlPFC) and dorsolateral (dlPFC) subregions, frontopolar (FPC), and orbitofrontal (OFC), (2) striatum including caudate nucleus (Cd), putamen (Pu), and nucleus accumbens (Acb), and (3) in midbrain, substantia nigra/ventral tegmental area (SN/VTA). *Pharmacokinetic modeling*: Using a cerebellar reference region, distribution volume ratios (DVRs) were calculated for the periods 20–60 min (D1) and 90–150 min (D2 and DAT) post-injection of radiotracer (Logan et al., 1996). The binding potential with respect to non-displaceable tracer, proportional to the available receptor concentration, was then given by BP_*ND*_ = DVR-1 (Innis et al., 2007).

### Statistical Analyses

The sensory scores for the SPS-M are described in detail in Schneider et al. (2008b). In this paper we used “sensory factor 1” as reported in Schneider et al. (2008b), hereafter referred to as “sensory score.” This variable represents the magnitude of the sensory response across the three stimuli (feather, cotton, brush) and failure to habituate to the feather and cotton ball. Hence, higher scores indicate higher sensory responsivity, and less habituation over trials.

Relationships between sensory score and binding of the three separate radiotracers measuring D1R, D2R, and DAT in the ROIs were analyzed by Pearson correlations. We examined scatterplots separately by experiment, prior to combining the two experiments, and the two experiments are shown as distinct symbols in Figures 2–4.

## Results

**FIGURE 1:**
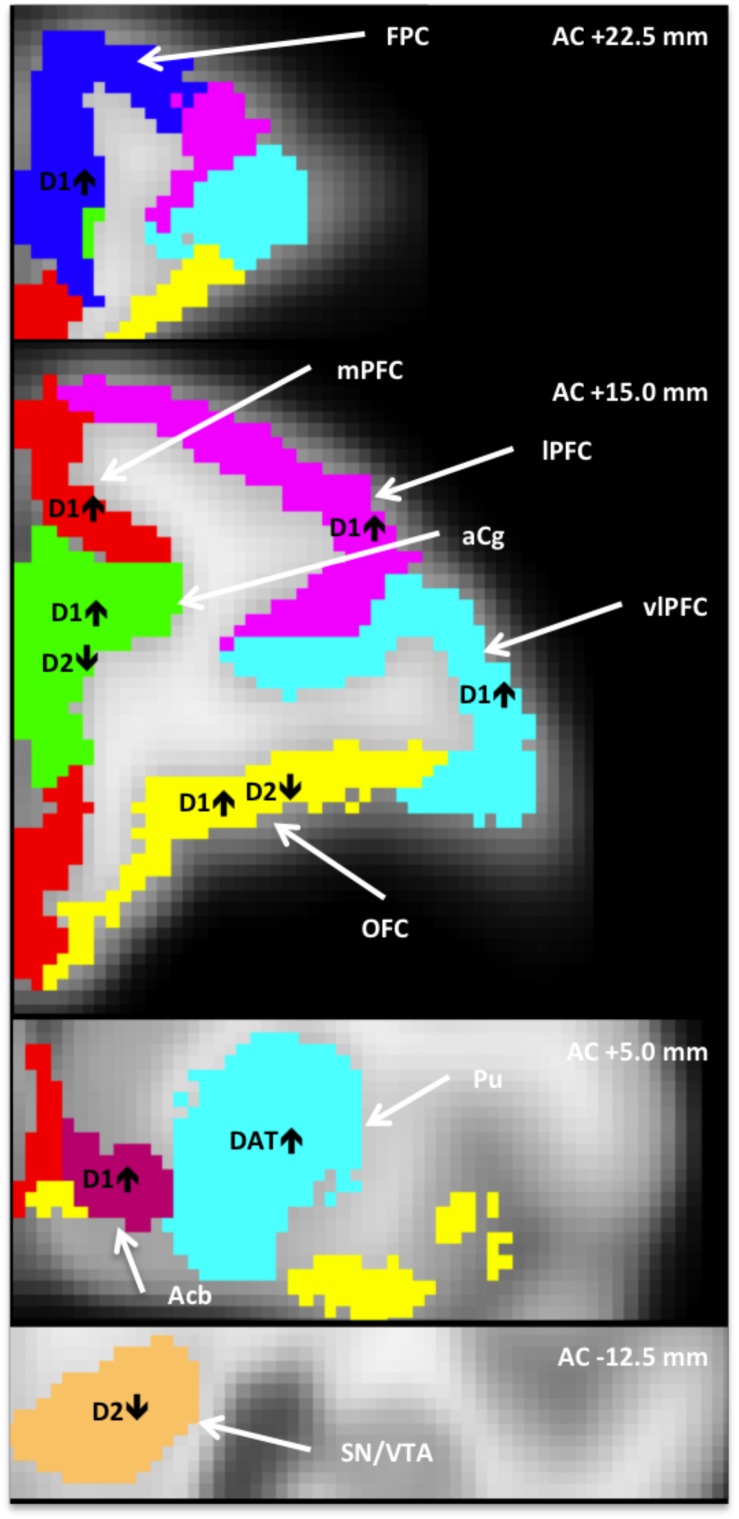
Anatomically defined regions overlaid on an MRI template (Moirano et al., 2018), in which dopaminergic measures correlated with heightened tactile responsivity (HTR). AC+/–, position relative to anterior commissure; Acb, nucleus accumbens; aCg, anterior cingulate; FPC, frontopolar cortex; lPFC, lateral PFC; mPFC, medial PFC; OFC, orbitofrontal cortex; vlPFC, ventrolateral PFC; PFC, prefrontal cortex; Pu, putamen; SN/VTA, substantia nigra/ventral tegmental area.

Table 1 shows the relations between binding potential for each of the three radiotracers and sensory scores for the brain ROI’s examined here (PFC, striatum, and midbrain). The regions with significant correlations are summarized in Figure 1. We present the results by each aspect of the DAergic system in turn, D1R, D2R, and DAT.

**TABLE 1:** Pearson correlations between sensory score and binding of the three radiotracers listed by brain ROI.

**Binding target**	**D1R**		**D2R**		**DAT**	
Radiotracer	[^11^C]SCH 23390		[^18^F]fallypride		[^18^F]FECNT	
Sample size	*N* = 64, *df* = 62		*N* = 46, *df* = 44		*N* = 73, *df* = 71	
Age (years) at scan: Mean (sd)	13.06 (1.62)		14.50 (3.89)		12.85 (1.44)	
	Pearson correlation (95% conf. int.)	Raw *p*-value (p adjusted by FDR)	Pearson correlation (95% conf. int.)	Raw *p*-value (p adjusted by FDR)	Pearson correlation (95% conf. int.)	Raw *p*-value (p adjusted by FDR)
**PFC**	**0.30 (0.06,0.51)**	**0.016 (0.032)**	−0.19 (−0.45,0.11)	0.214 (0.408)	0.06 (−0.18,0.28)	0.640 (0.800)
lPFC	**0.25 (0.01,0.47)**	**0.045 (0.052)**	−0.08 (−0.36,0.22)	0.611 (0.698)	No sig. binding	
vlPFC	**0.27 (0.03,0.48)**	**0.031 (0.041)**	−0.17 (−0.44,0.13)	0.255 (0.408)	0.07 (−0.16,0.30)	0.532 (0.800)
dlPFC	0.23 (−0.02,0.45)	0.069 (0.069)	−0.01 (−0.30,0.28)	0.964 (0.964)	No sig. binding	
mPFC	**0.32 (0.08,0.52)**	**0.011 (0.032)**	−0.21 (−0.47,0.09)	0.166 (0.408)	0.14 (−0.10,0.36)	0.248 (0.655)
aCg	**0.27 (0.03,0.49)**	**0.028 (0.041)**	**−0.30 (−0.54, −0.01)**	**0.043 (0.178)**	0.13 (−0.10,0.35)	0.262 (0.655)
FPC	**0.30 (0.06,0.51)**	**0.016 (0.032)**	−0.14 (−0.41,0.16)	0.365 (0.487)	No sig. binding	
OFC	**0.30 (0.06,0.51)**	**0.016 (0.032)**	−**0.30 (−0.54, −0.01)**	**0.045 (0.178)**	−0.02 (−0.25,0.21)	0.890 (0.890)
**Striatum**	0.22 (−0.03,0.44)	0.081 (0.108)	−0.25 (−0.50, 0.05)	0.098 (0.140)	0.23 (−0.00,0.44)	0.053 (0.105)
Acb	**0.27 (0.02,0.48)**	**0.032 (0.108)**	−0.22 (−0.54, −0.01) ^*^	0.154 (0.154)	0.16 (−0.08,0.37)	0.187 (0.187)
Cd	0.23 (−0.02,0.45)	0.068 (0.108)	−0.24 (−0.50,0.05)	0.103 (0.140)	0.19 (−0.04,0.41)	0.102 (0.136)
Pu	0.20 (−0.05,0.42)	0.116 (0.116)	−0.24 (−0.50,0.05)	0.105 (0.140)	**0.24 (0.01,0.45)**	**0.042 (0.105)**
**SN/VTA**	0.12 (−0.13,0.36)	0.349	**−0.30 (−0.54, −0.01)**	**0.043**	0.05 (−0.18,0.28)	0.686

Correlations with unadjusted *p* < 0.05 in bold. ^*^N = 45, one outlier removed. FDR denotes p-adjustment by false discovery rate within each major brain region (PFC and Striatum) by radiotracer.

### D1R Binding

**FIGURE 2:**
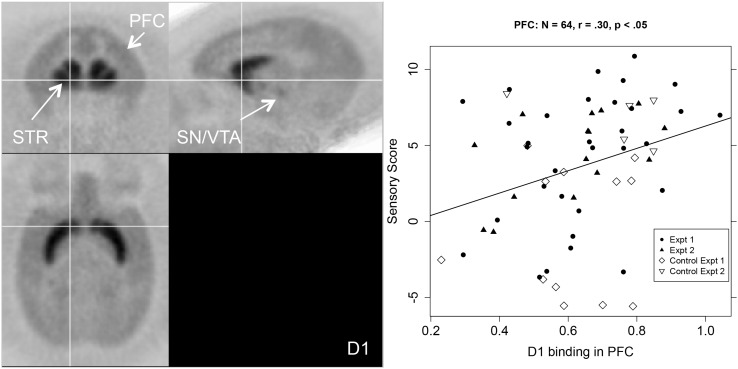
Left-hand panel: Representative average PET image of [^11^C]SCH 23390 uptake based on a subset of the subjects (*n* = 12). Right-hand panel: Scatterplot of the relationship between PFC D1R binding (BP_*ND*_, *x*-axis) and sensory score (*y*-axis) for both experiments, with regression line.

As shown in the first column of Table 1, the relationship between sensory score and D1R binding potential in the PFC was significant for the whole PFC (*r* = 0.30, *p* < 0.05), and also for all of the more detailed PFC ROIs except the dlPFC. Figure 2 shows a PET image of typical D1R binding potential in PFC in the left-hand panel. The right-hand panel shows the scatterplot of the relation between sensory score and D1R binding potential in PFC, along with the linear regression. Outside of PFC, the Acb also showed a significant positive correlation between D1R binding potential and sensory score.

### D2R Binding

**FIGURE 3:**
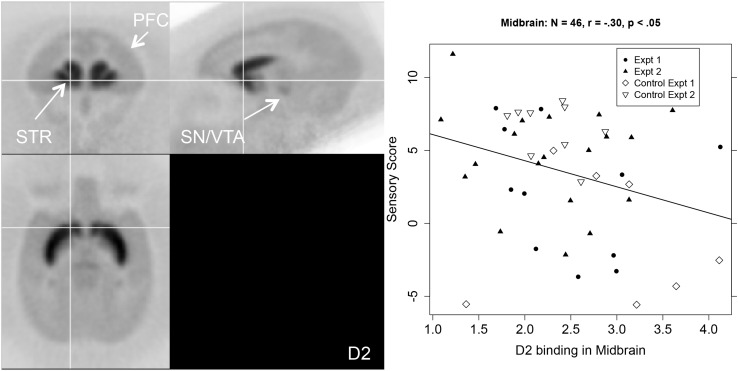
Left-hand panel: Representative average PET image of [^18^F]fallypride uptake based on a subset of the subjects (*n* = 20). Right-hand panel: Scatterplot of the relationship between midbrain (SN/VTA) D2R binding (BP_*ND*_, *x*-axis) and sensory score (*y*-axis) with regression line.

The middle columns of Table 1 show that sensory score was negatively correlated with D2R binding potential in the aCg, OFC, and SN/VTA. Figure 3 shows a PET image of typical D2R binding potential in the left-hand panel. The right-hand panel of Figure 3 shows the scatterplot of the relation between sensory score and D2R binding potential in midbrain, along with the linear regression.

### DAT Binding

**FIGURE 4:**
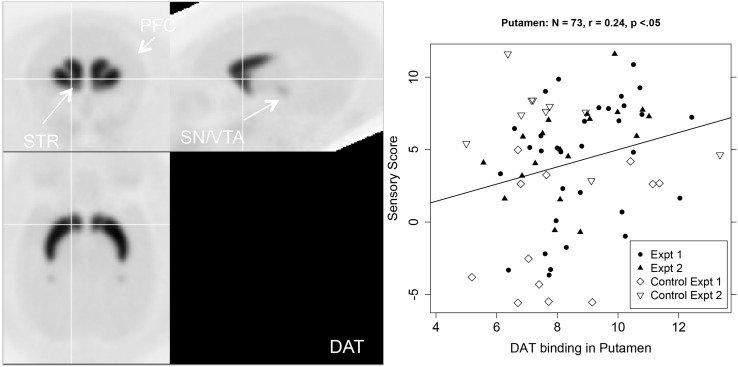
Left-hand panel: Representative average PET image of [^18^F]FECNT uptake based on a subset of the subjects (*n* = 12). Right-hand panel: Scatterplot of the relationship between DAT binding in putamen (BP_*ND*_, *x*-axis) and sensory score (*y*-axis), with regression line.

The right hand two columns of Table 1 show that sensory score was unrelated to DAT binding potential, except for a significant positive correlation in the putamen, with a trend in the whole striatum. Figure 4 shows a PET image of typical DAT binding potential, and the scatterplot for the significant relation between sensory score and DAT binding potential in putamen. As might be expected (Converse et al., 2013), DAT showed no significant binding (i.e., binding potential not significantly greater than zero) for three of the cortical areas (the lPFC, dlPFC, and the FPC).

## Discussion

A unique contribution of our study is that, to our knowledge, this is the first study to use PET neuroimaging to interrogate underlying DA neurotransmitter function for possible associations with heightened tactile responsivity (HTR) to non-noxious stimuli in monkeys. Below we integrate the results of our study with the research and clinical findings on children with SPD, the literature on the functions of DA in various areas of the brain, and the functional significances of the brain *pathways* to which our results pertain. We also relate the results to concepts in the literature regarding optimal D1R levels, and the complementarity and distinct functions of D1Rs, D2Rs, and DAT.

### PFC

The first findings from this study are that in the PFC, including the mPFC, vlPFC, frontopolar PFC, and aCg, HTR is related to *elevated* D1R, and *reduced* D2R availability in OFC and aCg. PFC is an evolutionarily advanced structure that projects to other cortical and subcortical areas to modulate many sensory and affective functions (Fuster, 2009; Arnsten et al., 2012). The PFC is a major component of a cortical network that links stimulus perception and action in order for the organism to adaptively respond to continuously changing environments (Haller et al., 2018). PET studies of adults are consistent with conclusions from the animal literature that D1Rs in PFC are involved in motor function, reward mechanisms, learning and working memory (Goldman-Rakic, 1995; Beaulieu and Gainetdinov, 2011), behavioral functions that are challenging for many individuals with SPD (Ayres, 1969).

In our study, *both* elevated D1R availability and reduced D2R availability in OFC and aCg were related to HTR. OFC, a primary component of PFC, has extensive connections with sensory areas as well as limbic regions involved in goal-directed decision making, emotional processing, and flexible responding based on reward value (Iversen and Mishkin, 1970; Damasio, 1996; Schoenbaum et al., 2002; McAlonan and Brown, 2003; Gourley et al., 2016). OFC signals expectations of future outcomes and can heighten anticipatory anxiety by inflating prediction of threat (Grupe and Nitschke, 2013; Stalnaker et al., 2015).

In mPFC, including the aCg, HTR was related to increased D1R availability. The mPFC is considered to be a limbic forebrain area that supports not only sensorimotor gating (Graham, 1975; Koch and Bubser, 1994; Ellenbroek et al., 1996; Lacroix et al., 2000; Swerdlow et al., 2001; Toth et al., 2017), but also complex goal-directed behaviors, cognitive flexibility, attention, emotion, and “self-referential” emotion processing (Seamans et al., 1998; Damasio, 2003; Dalley et al., 2004; Northoff and Heinzel, 2006; Ragozzino, 2007; Paine et al., 2011; Cassaday et al., 2014; Pezze et al., 2014). Thus, increased D1R availability in aCg and mPFC is consistent with research indicating that children with SPD have difficulties in mood and emotion regulation, attention, and spatial memory, as well as sensorimotor gating.

Lastly, for our cortical results, our study showed that HTR was related to elevated D1R availability in ventrolateral and frontopolar PFC. These areas are considered important for information integration and response selection, coupling stimulus perception with action, and thereby enabling flexible responding (Burgess, 2011). Flexible responding can pose difficulties in children with SPD (Ayres and Robbins, 1979).

To date, it appears there are no studies of D1R availability in PFC in children or adults with SPD. Highlighting the importance of D1R in behavioral regulation, studies in patients with various psychiatric diagnoses have shown either elevation or reduction of D1R availability in frontal cortex. Psychiatric diagnoses studied include seasonal affective disorder (Plaven-Sigray et al., 2017), schizophrenia (Kosaka et al., 2010), and drug naïve patients with schizophrenia (Abi-Dargham et al., 2012), and see (Cervenka, 2019). Studies are clearly needed to examine D1R levels in individuals with SPD who do not have other psychiatric disorders.

### Striatum

The striatum, which includes the putamen, Acb, and caudate nucleus, is a subcortical structure that has a critical role in motor control, cognition, behavioral flexibility, and associative behaviors, functions in which children with SPD are often challenged (Miller et al., 2017). We found that increased DAT in putamen and increased D1R in the Acb were related to HTR. DAT in striatum is considered to be important for maintaining dopaminergic tone, that is, homeostatic levels of synaptic DA (Volkow et al., 2002). It is possible that increased DAT binding potential in putamen is associated with HTR in the present study in part because of problematic homeostatic DA functions. Our present results are consistent with our previous publication on Experiment 1 that showed that overall magnitude of sensory responsivity and habituation to repeated tactile stimulation were related to DAT binding potential in striatum (Converse et al., 2013).

The Acb, a main structure of the ventral striatum, is also a major component of a pain regulation pathway to the PFC, as well as having involvement in reward processing and substance use (Becerra and Borsook, 2008). An interesting issue relevant to our finding of a relation between HTR and increased D1R availability in Acb concerns the relationship between DA function and social behavior. It is well documented that children with SPD often have social difficulties (Ben-Sasson et al., 2013). Early social interactions in mammals involve nursing and parental care, in which the oxytocin-mesolimbic DA systems play an important role (Numan and Sheehan, 1997). Animal and human studies indicate that processing of social-emotional stimuli occurs in brain regions that also process reward, including the Acb (Robinson et al., 2002). Parental caregiving, social play and sexual behaviors are immensely rewarding for both humans (Izuma et al., 2008; Spreckelmeyer et al., 2009) and animals (Trezza et al., 2011) leading to pleasure, well-being, and associative learning (Berridge and Kringelbach, 2008). An increase in DA signaling, particularly in the Acb reward system, has been shown in high licking/grooming rodent mothers, accompanied by increased levels of D1 and D3 receptors in Acb (Champagne et al., 2004), whereas maternal neglect is associated with dysregulation of DA transmission (Numan and Sheehan, 1997). The social linkages of DA in Acb also depend on neural connections to the midbrain, especially the VTA (Gunaydin et al., 2014).

### Midbrain

Dopamine cell bodies are located in the midbrain in the substantia nigra (SN) and the ventral tegmentum (VTA), and they project to the striatum and PFC. D2Rs in SN/VTA serve as auto-receptors in a negative feedback loop to moderate dopaminergic signaling (Ford, 2014). In this study, reduced D2R availability in SN/VTA was associated with HTR. Interestingly, in mice, activation of DA neurons in the VTA that project to Acb enhanced social interaction; this increase in social interaction was blocked by a D1R antagonist infused into the Acb (Gunaydin et al., 2014). Moreover, increased D1R signaling restored social interaction and hedonic behaviors, while inhibition of VTA DA neurons projecting to Acb enhanced depressive-like behaviors (Francis et al., 2015). Given these rodent findings, it is plausible that the coupling of altered DA function with HTR in our study, which was pronounced in the DA-mediated reward and pain pathways, might underpin the social challenges that many children with SPD show.

### Implications for Functional Pathways Across Midbrain, Striatum and PFC

As mentioned in the introduction, research on children using DTI has identified a number of pathways that appear to be disrupted in SPD, including some limited evidence for reduced connectivity in frontal tracts, as well as disruption of posterior-located sensory projection areas (Owen et al., 2013; Chang et al., 2014, 2016). These findings in children suggest the importance of further research on the roles of neurotransmitter functioning in brain connectivity in SPD.

Highly relevant to SPD is the strong functional connectivity of PFC and Acb, a critical pathway that regulates both sensory and affective elements of pain (Koob and Volkow, 2010; Zhou et al., 2018). Projections from PFC to Acb have also been shown to inhibit *both* acute and chronic pain behaviors in rodents (Lee et al., 2015; Martinez et al., 2017). In rats, disruption of this pathway heightens nociceptive sensitivity and enhances aversive responses to pain stimuli (Zhou et al., 2018), whereas excitation of this pathway reduces pain behaviors and inhibits withdrawal responses (Cooper, 1975; Hardy, 1985).

Research supports the idea that the cortico-limbic pathway (mPFC, including the aCg, and OFC) provides top-down regulation of sensory and affective processes via the PFC-Acb pathway. The bottom-up midbrain-striato-frontal pathway (VTA-Acb-PFC) provides the motivation or drive for action, and both the VTA-Acb projection and the VTA-mPFC projections have been shown be directly involved in reward (Han et al., 2017). We found opposing effects of D1R availability and D2R availability in both aCg and OFC. The balance between these two complex and interdependent pathways, the top-down cognitive control PFC-Acb pathway and the bottom-up motivational or drive VTA-Acb-PFC pathway, is considered important for successful goal-directed behavior and mood (Casey and Jones, 2010; Russo and Nestler, 2013). Imbalances in the interactions between these two systems can yield behaviors biased toward the subcortical motivational system, including exaggerated reactivity to motivational stimuli and sensation-seeking. Such imbalances are thought to be as a consequence of delayed or altered development of the top-down PFC regulatory system (Casey et al., 2008; Casey and Jones, 2010). Sensation-seeking and risky behavior are characteristics often linked to SPD (Miller et al., 2017). Taken together, our current data support the notion that D1R:D2R mediated imbalances in the PFC-Acb reward and pain regulation pathway, could involve reduction of the PFC top-down control. In turn, this imbalance could cascade into the sensory over-responsive phenotype of SPD along with other cognitive and affective behaviors.

### Complementarity of D1R and D2R Functions

Our findings are also in line with evidence that D1R and D2R have distinct and often opposing functions. For example, D1 and D2 receptors exert opposite effects in locomotion and its spatial distribution, as well as snout contact, mouthing, and grooming (Eilam et al., 1991, 1992). So, it is not surprising, for example, that D1R versus D2R knock-out mice show opposite phenotypes in cognitive and motor tasks (Nakamura et al., 2014). Increased D1R receptor availability, with no change in D2R receptor availability, alters the ratio of D1R:D2R signaling toward D1R, which is thought to contribute to risk for both addiction and hyperactivity (Robison et al., 2018). Interestingly, optimal cognition follows an inverted U-shaped function such that either inadequate or excessive D1R stimulation can erode cognition while moderate levels can enhance function (Williams and Goldman-Rakic, 1995; Granon et al., 2000; Vijayraghavan et al., 2007). Optimal D1R stimulation is thought to gate out “noisy input” from nearby connections through a variety of mechanisms (see Arnsten et al., 2012, 2015).

A further concept relevant to the potential role of DA in SPD is that neurotransmitter activity modulated via the D1 versus the D2 receptor subtypes may affect the activity of thalamocortical neurons that relay sensory information from the periphery to the sensory cortex and other brain areas. Different firing patterns appear to be associated with behavioral state changes and, in turn, influence behavior (Govindaiah et al., 2010). Taken together, our findings of elevated D1R availability and reduced D2R availability in OFC and aCg suggest that alteration of D1R could give rise to downstream effects (altered DA receptors in other regions) that persist and influence HTR symptoms.

### Possible Developmental Origins of the Association of Heightened Tactile Responsivity and DA

More detailed elucidation of the mechanisms behind the association of DAergic functions and HTR is needed. One possibility is that abnormal DA system development may alter synaptic plasticity as well as structural connectivity during the neural development of the ventrolateral and dorsolateral PFC. Zhou et al. (2012) contend that D1R up-regulation is one source of abnormalities in synaptic plasticity which, in turn, can underlie neurobehavioral deficits. Conversion of long-term potentiation (LTP) to long-term depression (LTD) in synapses takes place around the postnatal third week in the rat (Partridge et al., 2000) with the DA system playing a critical role in this transformation (Tang et al., 2002). LTP first appears when synapses are beginning to function in striatum (Partridge et al., 2000). LTD emerges later to better calibrate synapses for skilled movement and sequencing of behavior (Di Filippo et al., 2009). Zhou et al. (2012) found that high dose prenatal alcohol exposure (6 g/kg/d, gestation days 7 through 20), resulted in the emergence of LTP instead of LTD at postnatal day 30 by altering D1R and D2R functions in the dorsolateral striatum in male rodent offspring. Thus, it is possible that the altered D1R, D2R, and DAT functions related to HTR detected in our longitudinal studies might be the outcome of altered processes during early development, perhaps especially synaptic plasticity driven by DA. Alteration of these early life neurodevelopmental functions could also lead to possible mis-wiring of neural connections and result in disrupted neurobehavioral outcomes, including SPD.

### Limitations

This paper focused only on DAergic function. However, there are other neurotransmitters such as serotonin, glutamate, and GABA, that could interact with DA and contribute to the progression and manifestation of SPD. For example, serotonin can alter DAergic signaling and transmission by activating DA neurons in VTA and Acb (Campbell et al., 1996). Despite this limitation of studying only the DA system, the use of *in vivo* PET is an important strength in this study because it provides quantification of markers of brain neurotransmission in order to examine how DA function correlates with the behavioral phenotype of HTR. Also, the use of 3 radioligands, [^11^C]SCH 23390, [^18^F]fallypride, and [^18^F]FECNT, affords the opportunity to examine D1R, D2R, and DAT availability in separate scans in the same subject.

As in most non-human primate research, the sample size here is limited. A limited sample size is also a common problem in neuroscience research with humans, particularly so in neuroimaging studies with special populations. However, our minimum of 46 subjects is relatively large compared with other primate PET studies. Moreover, a limitation is that the prenatal conditions differed somewhat across the two experiments combined here, and were not analyzed in this paper. In both experiments, monkeys were derived from mothers that, in pre-screening, would voluntarily consume moderate-dose alcohol. These females were then randomly assigned to consume alcohol during specific gestation periods, alone or in combination with mild prenatal stress exposure, compared with randomly assigned controls. We did not include the prenatal treatment findings in this paper because they have been reported elsewhere (Schneider et al., 2008a, 2009, 2017; Converse et al., 2013, 2014).

## Conclusion

The results of the present study are the first to demonstrate *in vivo* that altered D1R, D2R, and DAT availability in the midbrain-cortico-striatal network has a relationship to heightened tactile responsivity in non-human primates. In particular, our evidence supports the likely role of heightened D1R availability in the PFC, including the OFC (cortical) and Acb (subcortical) reward and pain regulation pathways as potential contributors to the neural substrate for SPD. Overall, the results provide support for the hypothesis that imbalances in cortical/subcortical circuitries including OFC-Acb reward circuitry, in which DA signaling via D1R and D2Rs is critical, may be key in the pathophysiology of SPD.

A final noteworthy issue concerns the potential of environmental enrichment as a treatment for DA-related molecular and behavioral effects. In rodents, environmental enrichment has been shown to reduce D1R expression in PFC and striatum (Del Arco et al., 2007; Gill et al., 2013) and decrease DAT in PFC (Kim et al., 2016), producing long-lasting functional changes in mesolimbic DA transmission (Darna et al., 2015). In addition, numerous beneficial behavioral effects have resulted from environmental enrichment in rodents (Fernandez-Teruel et al., 2002; Galani et al., 2007; Green et al., 2010; Harati et al., 2013). In non-human primates, social enrichment has been shown to reverse the effects of early life social isolation and lack of touch (Suomi et al., 1972).

Animal studies are needed to examine sensitive windows of the development of DA pathways to improve treatment efficacy and therefore diminish the psychological cost of SPD on individuals, their families, and the burdens on society (Reynolds et al., 2010). Human studies are needed to examine whether interventions to reduce tactile sensitivities and improve developmental outcomes in young children, such as sensory-integration occupational therapy (Schaaf et al., 2018), could improve DA function as well as SPD-related behaviors such as cognitive control, mood regulation, and adaptive life skills.

## Ethics Statement

This study was carried out in accordance with recommendations of the USDA and NIH regarding animal welfare. Protocols were approved by the University of Wisconsin–Madison, Institutional Animal Care and Use Committee.

## Author Contributions

MS, CM, BC, OD, JH, RN, and AC designed the experiments. MS, EA, TB, BC, OD, JE, JL, LR, DM, RN, and AC performed the experiments. MS, CM, JH, JM, and AC contributed to the data analysis. MS, CM, and AC contributed to the writing of the manuscript.

## Conflict of Interest Statement

The authors declare that the research was conducted in the absence of any commercial or financial relationships that could be construed as a potential conflict of interest.

## References

[B1] Abi-Dargham A. Xu X. Thompson J. L. Gil R. Kegeles L. S. Urban N. (2012). Increased prefrontal cortical D(1) receptors in drug naive patients with schizophrenia: a PET study with [11C]NNC112. *J. Psychopharmacol.* 26 794–805. 10.1177/0269881111409265 21768159

[B2] Ahn R. R. Miller L. J. Milberger S. McIntosh D. N. (2004). Prevalence of parents’ perceptions of sensory processing disorders among kindergarten children. *Am. J. Occup. Ther.* 58 287–293. 10.5014/ajot.58.3.287 15202626

[B3] American Psychiatric Association (2013). *Diagnostic and Statistical Manual of Mental Disorders*, 5th Edn Washington, DC: American Psychiatric Publishing.

[B4] Arnsten A. F. T. Wang M. Paspalas C. D. (2015). Dopamine’s actions in primate prefrontal cortex: challenges for treating cognitive disorders. *Pharmacol. Rev.* 67 681–696. 10.1124/pr.115.010512 26106146 PMC4485014

[B5] Arnsten A. F. T. Wang M. J. Paspalas C. D. (2012). Neuromodulation of thought: flexibilities and vulnerabilities in prefrontal cortical network synapses. *Neuron* 76 223–239. 10.1016/j.neuron.2012.08.038 23040817 PMC3488343

[B6] Ayres A. J. (1964). Tactile functions. Their relation to hyperactive and perceptual motor behavior. *Am. J. Occup. Ther.* 18 6–11. 14116444

[B7] Ayres A. J. (1969). Deficits in sensory integration in educationally handicapped children. *J. Learn. Disabil.* 2 160–168. 10.1177/002221946900200307

[B8] Ayres A. J. (1972). Types of sensory integrative dysfunction among disabled learners. *Am. J. Occup. Ther.* 26 13–18. 5008164

[B9] Ayres A. J. Robbins J. (1979). *Sensory Integration and the Child.* Los Angeles, CA: Western Psychological Services.

[B10] Baranek G. T. Berkson G. (1994). Tactile defensiveness in children with developmental disabilities: responsiveness and habituation. *J. Autism Dev. Disord.* 24 457–471. 10.1007/bf02172128 7961330

[B11] Bar-Shalita T. Vatine J.-J. Parush S. (2008). Sensory modulation disorder: a risk factor for participation in daily life activities. *Dev. Med. Child Neurol.* 50 932–937. 10.1111/j.1469-8749.2008.03095.x 19046186

[B12] Beaulieu J.-M. Gainetdinov R. R. (2011). The physiology, signaling, and pharmacology of dopamine receptors. *Pharmacol. Rev.* 63 182–217. 10.1124/pr.110.002642 21303898

[B13] Becerra L. Borsook D. (2008). Signal valence in the nucleus accumbens to pain onset and offset. *Eur. J. Pain* 12 866–869. 10.1016/j.ejpain.2007.12.007 18226937 PMC2581873

[B14] Ben-Sasson A. Carter A. S. Briggs-Gowan M. J. (2009). Sensory over-responsivity in elementary school: prevalence and social-emotional correlates. *J. Abnorm. Child Psychol.* 37 705–716. 10.1007/s10802-008-9295-8 19153827 PMC5972374

[B15] Ben-Sasson A. Soto T. W. Martinez-Pedraza F. Carter A. S. (2013). Early sensory over-responsivity in toddlers with autism spectrum disorders as a predictor of family impairment and parenting stress. *J. Child Psychol. Psychiatry* 54 846–853. 10.1111/jcpp.12035 23336424 PMC3636173

[B16] Berridge K. C. Kringelbach M. L. (2008). Affective neuroscience of pleasure: reward in humans and animals. *Psychopharmacology* 199 457–480. 10.1007/s00213-008-1099-6 18311558 PMC3004012

[B17] Burgess P. W. (2011). Frontopolar cortex: constraints for theorizing. *Trends Cogn. Sci.* 15:242;authorreply243. 10.1016/j.tics.2011.04.006 21571576

[B18] Campbell A. D. Kohl R. R. McBride W. J. (1996). Serotonin-3 receptor and ethanol-stimulated somatodendritic dopamine release. *Alcohol* 13 569–574. 10.1016/s0741-8329(96)00069-9 8949951

[B19] Carter A. S. Ben-Sasson A. Briggs-Gowan M. J. (2011). Sensory over-responsivity, psychopathology, and family impairment in school-aged children. *J. Am. Acad. Child Adolesc. Psychiatry* 50 1210–1219. 10.1016/j.jaac.2011.09.010 22115142 PMC5228273

[B20] Cascio C. J. Moore D. McGlone F. (2019). Social touch and human development. *Dev. Cogn. Neurosci.* 35 5–11. 10.1016/j.dcn.2018.04.009 29731417 PMC6968965

[B21] Casey B. J. (2001). “Disruption of inhibitory control in developmental disorders: a mechanistic model of implicated fronto-striatal circuitry,” in *Mechanisms of Cognitive Development: Behavioral and Neural Perspectives*, eds McClelland J. L. Siegler R. S. (Mahwah, NJ: Erlbaum), 327–349.

[B22] Casey B. J. Jones R. M. (2010). Neurobiology of the adolescent brain and behavior: implications for substance use disorders. *J. Am. Acad. Child Adolesc. Psychiatry* 49 1189–1201. 10.1016/j.jaac.2010.08.017 21093769 PMC3099425

[B23] Casey B. J. Jones R. M. Hare T. A. (2008). The adolescent brain. *Ann. N. Y. Acad. Sci.* 1124 111–126. 10.1196/annals.1440.010 18400927 PMC2475802

[B24] Cassaday H. J. Nelson A. J. D. Pezze M. A. (2014). From attention to memory along the dorsal-ventral axis of the medial prefrontal cortex: some methodological considerations. *Front. Syst. Neurosci.* 8:160. 10.3389/fnsys.2014.00160 25249948 PMC4157611

[B25] Cervenka S. (2019). PET radioligands for the dopamine D1-receptor: application in psychiatric disorders. *Neurosci. Lett.* 691 26–34. 10.1016/j.neulet.2018.03.007 29518542

[B26] Champagne F. A. Chretien P. Stevenson C. W. Zhang T. Y. Gratton A. Meaney M. J. (2004). Variations in nucleus accumbens dopamine associated with individual differences in maternal behavior in the rat. *J. Neurosci.* 24 4113–4123. 10.1523/JNEUROSCI.5322-03.2004 15115806 PMC6729285

[B27] Chang Y.-S. Gratiot M. Owen J. P. Brandes-Aitken A. Desai S. S. Hill S. S. (2016). White matter microstructure is associated with auditory and tactile processing in children with and without sensory processing disorder. *Front. Neuroanat.* 9:169. 10.3389/fnana.2015.00169 26858611 PMC4726807

[B28] Chang Y.-S. Owen J. P. Desai S. S. Hill S. S. Arnett A. B. Harris J. (2014). Autism and sensory processing disorders: shared white matter disruption in sensory pathways but divergent connectivity in social-emotional pathways. *PLoS One* 9:e103038. 10.1371/journal.pone.0103038 25075609 PMC4116166

[B29] Converse A. K. Moore C. F. Holden J. E. Ahlers E. O. Moirano J. M. Larson J. A. (2014). Moderate-level prenatal alcohol exposure induces sex differences in dopamine d1 receptor binding in adult rhesus monkeys. *Alcohol. Clin. Exp. Res.* 38 2934–2943. 10.1111/acer.12575 25581649 PMC4293080

[B30] Converse A. K. Moore C. F. Moirano J. M. Ahlers E. O. Larson J. A. Engle J. W. (2013). Prenatal stress induces increased striatal dopamine transporter binding in adult nonhuman primates. *Biol. Psychiatry* 74 502–510. 10.1016/j.biopsych.2013.04.023 23726316 PMC3775901

[B31] Cooper S. J. (1975). Anaesthetisation of prefrontal cortex and response to noxious stimulation. *Nature* 254 439–440. 10.1038/254439a0 1118029

[B32] Dalley J. W. Cardinal R. N. Robbins T. W. (2004). Prefrontal executive and cognitive functions in rodents: neural and neurochemical substrates. *Neurosci. Biobehav. Rev.* 28 771–784. 10.1016/j.neubiorev.2004.09.006 15555683

[B33] Damasio A. (2003). Feelings of emotion and the self. *Ann. N. Y. Acad. Sci.* 1001 253–261. 14625365 10.1196/annals.1279.014

[B34] Damasio A. R. (1996). The somatic marker hypothesis and the possible functions of the prefrontal cortex. *Philos. Trans. R. Soc. Lond. B Biol. Sci.* 351 1413–1420. 10.1098/rstb.1996.0125 8941953

[B35] Darna M. Beckmann J. S. Gipson C. D. Bardo M. T. Dwoskin L. P. (2015). Effect of environmental enrichment on dopamine and serotonin transporters and glutamate neurotransmission in medial prefrontal and orbitofrontal cortex. *Brain Res.* 1599 115–125. 10.1016/j.brainres.2014.12.034 25536304 PMC4344853

[B36] Davies P. L. Gavin W. J. (2007). Validating the diagnosis of sensory processing disorders using EEG technology. *Am. J. Occup. Ther.* 61 176–189. 10.5014/ajot.61.2.176 17436840

[B37] DeJesus O. T. Van Moffaert G. J. Friedman A. M. (1987). Synthesis of [11C]SCH 23390 for dopamine D1 receptor studies. *Int J. Rad. Appl. Instrum. A* 38 345–348. 10.1016/0883-2889(87)90022-0 3038787

[B38] Del Arco A. Segovia G. Canales J. J. Garrido P. de Blas M. Garcia-Verdugo J. M. (2007). Environmental enrichment reduces the function of D1 dopamine receptors in the prefrontal cortex of the rat. *J. Neural Transm.* 114 43–48. 10.1007/s00702-006-0565-8 16955373

[B39] Di Filippo M. Picconi B. Tantucci M. Ghiglieri V. Bagetta V. Sgobio C. (2009). Short-term and long-term plasticity at corticostriatal synapses: implications for learning and memory. *Behav. Brain Res.* 199 108–118. 10.1016/j.bbr.2008.09.025 18948145

[B40] Eilam D. Clements K. V. Szechtman H. (1991). Differential effects of D1 and D2 dopamine agonists on stereotyped locomotion in rats. *Behav. Brain Res.* 45 117–124. 10.1016/s0166-4328(05)80077-4 1686397

[B41] Eilam D. Talangbayan H. Canaran G. Szechtman H. (1992). Dopaminergic control of locomotion, mouthing, snout contact, and grooming: opposing roles of D1 and D2 receptors. *Psychopharmacology* 106 447–454. 10.1007/bf02244813 1533720

[B42] Ellenbroek B. A. Budde S. Cools A. R. (1996). Prepulse inhibition and latent inhibition: the role of dopamine in the medial prefrontal cortex. *Neuroscience* 75 535–542. 10.1016/0306-4522(96)00307-7 8931016

[B43] Ellingsen D.-M. Leknes S. Loseth G. Wessberg J. Olausson H. (2015). The neurobiology shaping affective touch: expectation, motivation, and meaning in the multisensory context. *Front. Psychol.* 6:1986. 10.3389/fpsyg.2015.01986 26779092 PMC4701942

[B44] Fernandez-Teruel A. Gimenez-Llort L. Escorihuela R. M. Gil L. Aguilar R. Steimer T. (2002). Early-life handling stimulation and environmental enrichment: are some of their effects mediated by similar neural mechanisms? *Pharmacol. Biochem. Behav.* 73 233–245. 10.1016/s0091-3057(02)00787-6 12076742

[B45] Field T. Hernandez-Reif M. Diego M. Schanberg S. Kuhn C. (2005). Cortisol decreases and serotonin and dopamine increase following massage therapy. *Int. J. Neurosci.* 115 1397–1413. 10.1080/00207450590956459 16162447

[B46] Ford C. P. (2014). The role of D2-autoreceptors in regulating dopamine neuron activity and transmission. *Neuroscience* 282 13–22. 10.1016/j.neuroscience.2014.01.025 24463000 PMC4108583

[B47] Francis T. C. Chandra R. Friend D. M. Finkel E. Dayrit G. Miranda J. (2015). Nucleus accumbens medium spiny neuron subtypes mediate depression-related outcomes to social defeat stress. *Biol. Psychiatry* 77 212–222. 10.1016/j.biopsych.2014.07.021 25173629 PMC5534173

[B48] Fuster J. M. (2009). Cortex and memory: emergence of a new paradigm. *J. Cogn. Neurosci.* 21 2047–2072. 10.1162/jocn.2009.21280 19485699

[B49] Galani R. Berthel M.-C. Lazarus C. Majchrzak M. Barbelivien A. Kelche C. (2007). The behavioral effects of enriched housing are not altered by serotonin depletion but enrichment alters hippocampal neurochemistry. *Neurobiol. Learn. Mem.* 88 1–10. 10.1016/j.nlm.2007.03.009 17493843

[B50] Gill K. E. Beveridge T. J. R. Smith H. R. Porrino L. J. (2013). The effects of rearing environment and chronic methylphenidate administration on behavior and dopamine receptors in adolescent rats. *Brain Res.* 1527 67–78. 10.1016/j.brainres.2013.06.021 23806775 PMC3775327

[B51] Goldman-Rakic P. S. (1995). Cellular basis of working memory. [Review] [64 refs]. *Neuron* 14 477–485. 10.1016/0896-6273(95)90304-6 7695894

[B52] Goldman-Rakic P. S. Castner S. A. Svensson T. H. Siever L. J. Williams G. V. (2004). Targeting the dopamine D1 receptor in schizophrenia: insights for cognitive dysfunction. *Psychopharmacology* 174 3–16. 10.1007/s00213-004-1793-y 15118803

[B53] Gourley L. Wind C. Henninger E. M. Chinitz S. (2013). Sensory processing difficulties, behavioral problems, and parental stress in a clinical population of young children. *J. Child Fam. Stud.* 22 912–921. 10.1007/s10826-012-9650-9 24443636 PMC3891772

[B54] Gourley S. L. Zimmermann K. S. Allen A. G. Taylor J. R. (2016). The medial orbitofrontal cortex regulates sensitivity to outcome value. *J. Neurosci.* 36 4600–4613. 10.1523/JNEUROSCI.4253-15.2016 27098701 PMC4837686

[B55] Govindaiah G. Wang Y. Cox C. L. (2010). Dopamine enhances the excitability of somatosensory thalamocortical neurons. *Neuroscience* 170 981–991. 10.1016/j.neuroscience.2010.08.043 20801197

[B56] Graham F. K. (1975). Presidential address, 1974. The more or less startling effects of weak prestimulation. *Psychophysiology* 12 238–248. 10.1111/j.1469-8986.1975.tb01284.x 1153628

[B57] Granon S. Passetti F. Thomas K. L. Dalley J. W. Everitt B. J. Robbins T. W. (2000). Enhanced and impaired attentional performance after infusion of D1 dopaminergic receptor agents into rat prefrontal cortex. *J. Neurosci.* 20 1208–1215. 10.1523/jneurosci.20-03-01208.2000 10648725 PMC6774157

[B58] Green T. A. Alibhai I. N. Roybal C. N. Winstanley C. A. Theobald D. E. H. Birnbaum S. G. (2010). Environmental enrichment produces a behavioral phenotype mediated by low cyclic adenosine monophosphate response element binding (CREB) activity in the nucleus accumbens. *Biol. Psychiatry* 67 28–35. 10.1016/j.biopsych.2009.06.022 19709647 PMC2860655

[B59] Grupe D. W. Nitschke J. B. (2013). Uncertainty and anticipation in anxiety: an integrated neurobiological and psychological perspective. *Nat. Rev. Neurosci.* 14 488–501. 10.1038/nrn3524 23783199 PMC4276319

[B60] Gunaydin L. A. Grosenick L. Finkelstein J. C. Kauvar I. V. Fenno L. E. Adhikari A. (2014). Natural neural projection dynamics underlying social behavior. *Cell* 157 1535–1551. 10.1016/j.cell.2014.05.017 24949967 PMC4123133

[B61] Hall H. Sedvall G. Magnusson O. Kopp J. Halldin C. Farde L. (1994). Distribution of D1- and D2-dopamine receptors, and dopamine and its metabolites in the human brain. *Neuropsychopharmacology* 11 245–256. 10.1038/sj.npp.1380111 7531978

[B62] Haller M. Case J. Crone N. E. Chang E. F. King-Stephens D. Laxer K. D. (2018). Persistent neuronal activity in human prefrontal cortex links perception and action. *Nat. Hum. Behav.* 2 80–91. 10.1038/s41562-017-0267-2 29963646 PMC6022844

[B63] Han X. Jing M.-Y. Zhao T.-Y. Wu N. Song R. Li J. (2017). Role of dopamine projections from ventral tegmental area to nucleus accumbens and medial prefrontal cortex in reinforcement behaviors assessed using optogenetic manipulation. *Metab. Brain Dis.* 32 1491–1502. 10.1007/s11011-017-0023-3 28523568

[B64] Harati H. Barbelivien A. Herbeaux K. Muller M.-A. Engeln M. Kelche C. (2013). Lifelong environmental enrichment in rats: impact on emotional behavior, spatial memory vividness, and cholinergic neurons over the lifespan. *Age* 35 1027–1043. 10.1007/s11357-012-9424-8 22592932 PMC3705108

[B65] Hardy S. G. (1985). Analgesia elicited by prefrontal stimulation. *Brain Res.* 339 281–284. 10.1016/0006-8993(85)90093-9 4027626

[B66] Harlow H. F. Harlow M. K. (1962). The effect of rearing conditions on behavior. *Bull. Menninger Clin.* 26 213–224. 13904733

[B67] Hertenstein M. J. Verkamp J. M. Kerestes A. M. Holmes R. M. (2006). The communicative functions of touch in humans, nonhuman primates, and rats: a review and synthesis of the empirical research. *Genet., Soc. Gen. Psychol. Monogr.* 132 5–94. 10.3200/mono.132.1.5-94 17345871

[B68] Innis R. B. Cunningham V. J. Delforge J. Fujita M. Giedde A. Gunn R. N. (2007). Consensus nomenclature for *in vivo* imaging of reversibly binding radioligands. *J. Cereb. Blood Flow Metab.* 27 1533–1539. 10.1038/sj.jcbfm.9600493 17519979

[B69] Iversen S. D. Mishkin M. (1970). Perseverative interference in monkeys following selective lesions of the inferior prefrontal convexity. *Exp. Brain Res.* 11 376–386. 4993199 10.1007/BF00237911

[B70] Izuma K. Saito D. N. Sadato N. (2008). Processing of social and monetary rewards in the human striatum. *Neuron* 58 284–294. 10.1016/j.neuron.2008.03.020 18439412

[B71] Jenkinson M. Bannister P. Brady M. Smith S. (2002). Improved optimization for the robust and accurate linear registration and motion correction of brain images. *Neuroimage* 17 825–841. 10.1016/s1053-8119(02)91132-8 12377157

[B72] Kebabian J. W. Calne D. B. (1979). Multiple receptors for dopamine. *Nature* 277 93–96. 215920 10.1038/277093a0

[B73] Kim M.-S. Yu J. H. Kim C. H. Choi J. Y. Seo J. H. Lee M.-Y. (2016). Environmental enrichment enhances synaptic plasticity by internalization of striatal dopamine transporters. *J. Cereb. Blood Flow Metab.* 36 2122–2133. 10.1177/0271678X15613525 26661218 PMC5363660

[B74] Kisley M. A. Noecker T. L. Guinther P. M. (2004). Comparison of sensory gating to mismatch negativity and self-reported perceptual phenomena in healthy adults. *Psychophysiology* 41 604–612. 10.1111/j.1469-8986.2004.00191.x 15189483

[B75] Koch M. Bubser M. (1994). Deficient sensorimotor gating after 6-hydroxydopamine lesion of the rat medial prefrontal cortex is reversed by haloperidol. *Eur. J. Neurosci.* 6 1837–1845. 10.1111/j.1460-9568.1994.tb00576.x 7704295

[B76] Koob G. F. Volkow N. D. (2010). Neurocircuitry of addiction. *Neuropsychopharmacology* 35 217–238. 10.1038/npp.2009.110 19710631 PMC2805560

[B77] Kosaka J. Takahashi H. Ito H. Takano A. Fujimura Y. Matsumoto R. (2010). Decreased binding of [11C]NNC112 and [11C]SCH23390 in patients with chronic schizophrenia. *Life Sci.* 86 814–818. 10.1016/j.lfs.2010.03.018 20361984

[B78] Lacroix L. Spinelli S. Heidbreder C. A. Feldon J. (2000). Differential role of the medial and lateral prefrontal cortices in fear and anxiety. *Behav. Neurosci.* 114 1119–1130. 10.1037/0735-7044.114.6.1119 11142644

[B79] Lee M. Manders T. R. Eberle S. E. Su C. D’amour J. Yang R. (2015). Activation of corticostriatal circuitry relieves chronic neuropathic pain. *J. Neurosci.* 35 5247–5259. 10.1523/JNEUROSCI.3494-14.2015 25834050 PMC4380998

[B80] Logan J. Fowler J. S. Volkow N. D. Wang G. J. Ding Y. S. Alexoff D. L. (1996). Distribution volume ratios without blood sampling from graphical analysis of PET data. *J. Cereb. Blood Flow Metab.* 16 834–840. 10.1097/00004647-199609000-00008 8784228

[B81] Mailloux Z. Mulligan S. Roley S. S. Blanche E. Cermak S. Coleman G. G. (2011). Verification and clarification of patterns of sensory integrative dysfunction. *Am. J. Occup. Ther.* 65 143–151. 10.5014/ajot.2011.000752 21476361

[B82] Mangeot S. D. Miller L. J. McIntosh D. N. McGrath-Clarke J. Simon J. Hagerman R. J. (2001). Sensory modulation dysfunction in children with attention-deficit-hyperactivity disorder. *Dev. Med. Child Neurol.* 43 399–406. 11409829 10.1017/s0012162201000743

[B83] Martinez E. Lin H. H. Zhou H. Dale J. Liu K. Wang J. (2017). Corticostriatal regulation of acute pain. *Front. Cell. Neurosci.* 11:146. 10.3389/fncel.2017.00146 28603489 PMC5445115

[B84] Maruyama K. Shimoju R. Ohkubo M. Maruyama H. Kurosawa M. (2012). Tactile skin stimulation increases dopamine release in the nucleus accumbens in rats. *J. Physiol. Sci.* 62 259–266. 10.1007/s12576-012-0205-z 22411566 PMC10717409

[B85] McAlonan K. Brown V. J. (2003). Orbital prefrontal cortex mediates reversal learning and not attentional set shifting in the rat. *Behav. Brain Res.* 146 97–103. 10.1016/j.bbr.2003.09.019 14643463

[B86] Miller L. J. McIntosh D. N. McGrath J. Shyu V. Lampe M. Taylor A. K. (1999). Electrodermal responses to sensory stimuli in individuals with fragile X syndrome: a preliminary report. *Am. J. Med. Genet.* 83 268–279. 10.1002/(sici)1096-8628(19990402)83:4<268::aid-ajmg7>3.3.co;2-b 10208160

[B87] Miller L. J. Nielsen D. M. Schoen S. A. (2012). Attention deficit hyperactivity disorder and sensory modulation disorder: a comparison of behavior and physiology. *Res. Dev. Disabil.* 33 804–818. 10.1016/j.ridd.2011.12.005 22236629

[B88] Miller L. J. Nielsen D. M. Schoen S. A. Brett-Green B. A. (2009). Perspectives on sensory processing disorder: a call for translational research. *Front. Integr. Neurosci.* 3:22. 10.3389/neuro.07.022.2009 19826493 PMC2759332

[B89] Miller L. J. Schoen S. A. Mulligan S. Sullivan J. (2017). Identification of sensory processing and integration symptom clusters: a preliminary study. *Occup. Ther. Int.* 2017:2876080. 10.1155/2017/2876080 29348739 PMC5733937

[B90] Moirano J. M. Bezgin G. Y. Ahlers E. O. Kotter R. Converse A. K. (2018). Rhesus macaque brain atlas regions aligned to an MRI template. *Neuroinformatics* 17 295–306. 10.1007/s12021-018-9400-2 30291569 PMC6467493

[B91] Montagu A. (1984). The skin, touch, and human development. *Clin. Dermatol.* 2 17–26. 10.1016/0738-081x(84)90043-9 6545772

[B92] Muir D. W. (2002). Adult communications with infants through touch: the forgotten sense. *Hum. Dev.* 45 95–99. 10.1159/000048155

[B93] Mukherjee J. Yang Z. Y. Das M. K. Brown T. (1995). Fluorinated benzamide neuroleptics–III. Development of (S)-N-[(1-allyl-2-pyrrolidinyl)methyl]-5-(3-[18F]fluoropropyl)-2, 3-dimethoxybenzamide as an improved dopamine D-2 receptor tracer. *Nucl. Med. Biol.* 22 283–296. 10.1016/0969-8051(94)00117-3 7627142

[B94] Murali D. Barnhart T. E. Vandehey N. T. Christian B. T. Nickles R. J. Converse A. K. Dejesus O. T. (2013). An efficient synthesis of dopamine transporter tracer [(18)F]FECNT. *Appl. Radiat. Isot.* 72 128–132. 10.1016/j.apradiso.2012.10.010 23208243 PMC3540169

[B95] Nakamura T. Sato A. Kitsukawa T. Momiyama T. Yamamori T. Sasaoka T. (2014). Distinct motor impairments of dopamine D1 and D2 receptor knockout mice revealed by three types of motor behavior. *Front. Integr. Neurosci.* 8:56. 10.3389/fnint.2014.00056 25076876 PMC4097398

[B96] Northoff G. Heinzel A. (2006). First-person neuroscience: a new methodological approach for linking mental and neuronal states. *Philos. Ethics Humanit. Med.* 1:E3. 10.1186/1747-5341-1-3 16759399 PMC1459272

[B97] Numan M. Sheehan T. P. (1997). Neuroanatomical circuitry for mammalian maternal behavior. *Ann. N. Y. Acad. Sci.* 807 101–125. 10.1111/j.1749-6632.1997.tb51915.x 9071346

[B98] Owen J. P. Marco E. J. Desai S. Fourie E. Harris J. Hill S. S. Mukherjee P. (2013). Abnormal white matter microstructure in children with sensory processing disorders. *NeuroImage Clin.* 2 844–853. 10.1016/j.nicl.2013.06.009 24179836 PMC3778265

[B99] Paine T. A. Slipp L. E. Carlezon W. A. J. (2011). Schizophrenia-like attentional deficits following blockade of prefrontal cortex GABAA receptors. *Neuropsychopharmacology* 36 1703–1713. 10.1038/npp.2011.51 21490590 PMC3138652

[B100] Partridge J. G. Tang K. C. Lovinger D. M. (2000). Regional and postnatal heterogeneity of activity-dependent long-term changes in synaptic efficacy in the dorsal striatum. *J. Neurophysiol.* 84 1422–1429. 10.1152/jn.2000.84.3.1422 10980015

[B101] Pezze M. McGarrity S. Mason R. Fone K. C. Bast T. (2014). Too little and too much: hypoactivation and disinhibition of medial prefrontal cortex cause attentional deficits. *J. Neurosci.* 34 7931–7946. 10.1523/JNEUROSCI.3450-13.2014 24899715 PMC4044251

[B102] Plaven-Sigray P. Hedman E. Victorsson P. Matheson G. J. Forsberg A. Djurfeldt D. R. Cervenka S. (2017). Extrastriatal dopamine D2-receptor availability in social anxiety disorder. *Eur. Neuropsychopharmacol.* 27 462–469. 10.1016/j.euroneuro.2017.03.007 28377075

[B103] Ragozzino M. E. (2007). The contribution of the medial prefrontal cortex, orbitofrontal cortex, and dorsomedial striatum to behavioral flexibility. *Ann. N. Y. Acad. Sci.* 1121 355–375. 10.1196/annals.1401.013 17698989

[B104] Reynolds S. Lane S. J. (2008). Diagnostic validity of sensory over-responsivity: a review of the literature and case reports. *J. Autism Dev. Disord.* 38 516–529. 10.1007/s10803-007-0418-9 17917804

[B105] Reynolds S. Lane S. J. Richards L. (2010). Using animal models of enriched environments to inform research on sensory integration intervention for the rehabilitation of neurodevelopmental disorders. *J. Neurodev. Disord.* 2 120–132. 10.1007/s11689-010-9053-4 22127899 PMC3164047

[B106] Robinson D. L. Heien M. L. A. V. Wightman R. M. (2002). Frequency of dopamine concentration transients increases in dorsal and ventral striatum of male rats during introduction of conspecifics. *J. Neurosci.* 22 10477–10486. 10.1523/jneurosci.22-23-10477.2002 12451147 PMC6758730

[B107] Robison L. S. Swenson S. Hamilton J. Thanos P. K. (2018). Exercise reduces dopamine D1R and increases D2R in rats: implications for addiction. *Med. Sci. Sports Exerc.* 50 1596–1602. 10.1249/MSS.0000000000001627 29613999

[B108] Russo S. J. Nestler E. J. (2013). The brain reward circuitry in mood disorders. *Nat. Rev. Neurosci.* 14 609–625. 10.1038/nrn3381 23942470 PMC3867253

[B109] Schaaf R. C. Benevides T. Blanche E. I. Brett-Green B. A. Burke J. P. Cohn E. S. Schoen S. A. (2010). Parasympathetic functions in children with sensory processing disorder. *Front. Integr. Neurosci.* 4:4. 10.3389/fnint.2010.00004 20300470 PMC2839854

[B110] Schaaf R. C. Dumont R. L. Arbesman M. May-Benson T. A. (2018). Efficacy of occupational therapy using ayres sensory integration((R)): a systematic review. *Am. J. Occup. Ther.* 72:7201190010p1-7201190010p10. 10.5014/ajot.2018.028431 29280711

[B111] Scheggi S. De Montis M. G. Gambarana C. (2018). DARPP-32 in the orchestration of responses to positive natural stimuli. *J. Neurochem.* 147 439–453. 10.1111/jnc.14558 30043390

[B112] Schneider M. L. Moore C. F. Adkins M. Barr C. S. Larson J. A. Resch L. M. Roberts A. (2017). Sensory processing in rhesus monkeys: developmental continuity, prenatal treatment, and genetic influences. *Child Dev.* 88 183–197. 10.1111/cdev.12572 27338151 PMC5424533

[B113] Schneider M. L. Moore C. F. Becker E. F. (2001). Timing of moderate alcohol exposure during pregnancy and neonatal outcome in rhesus monkeys (*Macaca mulatta*). *Alcohol. Clin. Exp. Res.* 25 1238–1245. 10.1097/00000374-200108000-00021 11505056

[B114] Schneider M. L. Moore C. F. DeJesus O. T. Converse A. K. (2008a). Prenatal Stress Influences on Neurobehavior, Stress Reactivity, and Dopaminergic Function in Rhesus Macaques. in *Primate Models of Children’s Health and Developmental Disabilities* eds BorBacher T. M. Sackett G. P. Grant K. S. (New York, NY Academic Press)

[B115] Schneider M. L. Moore C. F. Gajewski L. L. Larson J. A. Roberts A. D. Converse A. K. Dejesus O. T. (2008b). Sensory processing disorder in a primate model: evidence from a longitudinal study of prenatal alcohol and prenatal stress effects. *Child Dev.* 79 100–113 10.1111/j.1467-8624.2007.01113.x 18269511 PMC4226060

[B116] Schneider M. L. Moore C. F. Larson J. A. Thiede A. J. Converse A. K. (2009). Relationship of stress responsivity, serotonin and dopamine function, and alcohol consumption in a prospective longitudinal primate study [abstract 211] in *Proceedigs of the Research Society on Alcoholism Annual Meeting*, San Diego, CA.

[B117] Schneider M. L. Roughton E. C. Lubach G. R. (1997). Moderate alcohol consumption and psychological stress during pregnancy induce attention and neuromotor impairments in primate infants. *Child Dev.* 68 747–759. 10.1111/j.1467-8624.1997.tb01959.x 29106730

[B118] Schoen S. A. Miller L. J. Brett-Green B. A. Nielsen D. M. (2009). Physiological and behavioral differences in sensory processing: a comparison of children with autism spectrum disorder and sensory modulation disorder. *Front. Integr. Neurosci.* 3:29. 10.3389/neuro.07.029.2009 19915733 PMC2776488

[B119] Schoenbaum G. Nugent S. L. Saddoris M. P. Setlow B. (2002). Orbitofrontal lesions in rats impair reversal but not acquisition of go, no-go odor discriminations. *Neuroreport* 13 885–890. 10.1097/00001756-200205070-00030 11997707

[B120] Seamans J. K. Floresco S. B. Phillips A. G. (1998). D1 receptor modulation of hippocampal-prefrontal cortical circuits integrating spatial memory with executive functions in the rat. *J. Neurosci.* 18 1613–1621. 10.1523/jneurosci.18-04-01613.1998 9454866 PMC6792740

[B121] Spreckelmeyer K. N. Krach S. Kohls G. Rademacher L. Irmak A. Konrad K. Grunder G. (2009). Anticipation of monetary and social reward differently activates mesolimbic brain structures in men and women. *Soc. Cogn. Affect. Neurosci.* 4 158–165. 10.1093/scan/nsn051 19174537 PMC2686229

[B122] Stalnaker T. A. Cooch N. K. Schoenbaum G. (2015). What the orbitofrontal cortex does not do. *Nat. Neurosci.* 18 620–627. 10.1038/nn.3982 25919962 PMC5541252

[B123] Suomi S. J. Harlow H. F. McKinney W. T. J. (1972). Monkey psychiatrists. *Am. J. Psychiatry* 128 927–932. 10.1176/ajp.128.8.927 4621656

[B124] Swerdlow N. R. Geyer M. A. Braff D. L. (2001). Neural circuit regulation of prepulse inhibition of startle in the rat: current knowledge and future challenges. *Psychopharmacology* 156 194–215. 10.1007/s002130100799 11549223

[B125] Tai Y. C. Chatziioannou A. Siegel S. Young J. Newport D. Goble R. N. (2001). Performance evaluation of the microPET P4: a PET system dedicated to animal imaging. *Phys. Med. Biol.* 46 1845–1862. 10.1088/0031-9155/46/7/308 11474929

[B126] Tai Y. C. Ruangma A. Rowland D. Siegel S. Newport D. F. Chow P. L. (2005). Performance evaluation of the microPET focus: a third-generation microPET scanner dedicated to animal imaging. *J. Nucl. Med.* 46 455–463. 15750159

[B127] Tang S. J. Reis G. Kang H. Gingras A.-C. Sonenberg N. Schuman E. M. (2002). A rapamycin-sensitive signaling pathway contributes to long-term synaptic plasticity in the hippocampus. *Proc. Natl. Acad. Sci. U.S.A* 99 467–472. 10.1073/pnas.012605299 11756682 PMC117583

[B128] Thompson W. R. Grusec J. (1970). Studies of early experience. In *Carmichael’s Manual of Child Psychology* 3rd Edn, ed. Mussen P. (New York, NY: Wiley and Sons) 565–654.

[B129] Toth A. Petyko Z. Galosi R. Szabo I. Karadi K. Feldmann A. Lenard L. (2017). Neuronal coding of auditory sensorimotor gating in medial prefrontal cortex. *Behav. Brain Res.* 326 200–208. 10.1016/j.bbr.2017.03.004 28284946

[B130] Trezza V. Campolongo P. Vanderschuren L. J. M. J. (2011). Evaluating the rewarding nature of social interactions in laboratory animals. *Dev. Cogn. Neurosci.* 1 444–458. 10.1016/j.dcn.2011.05.007 22436566 PMC6987553

[B131] Vijayraghavan S. Wang M. Birnbaum S. G. Williams G. V. Arnsten A. F. T. (2007). Inverted-U dopamine D1 receptor actions on prefrontal neurons engaged in working memory. *Nat. Neurosci.* 10 376–384. 10.1038/nn1846 17277774

[B132] Volkow N. D. Fowler J. S. Wang G. Ding Y. Gatley S. J. (2002). Mechanism of action of methylphenidate: insights from PET imaging studies. *J. Atten. Disord.* 6 (Suppl. 1), S31–S43. 12685517 10.1177/070674370200601s05

[B133] Wilbarger J. Gunnar M. Schneider M. Pollak S. (2010). Sensory processing in internationally adopted, post-institutionalized children. *J. Child Psychol. Psychiatry* 51 1105–1114. 10.1111/j.1469-7610.2010.02255.x 20738449 PMC3119572

[B134] Williams G. V. Goldman-Rakic P. S. (1995). Modulation of memory fields by dopamine D1 receptors in prefrontal cortex. *Nature* 376 572–575. 10.1038/376572a0 7637804

[B135] Williams K. L. Kirby A. V. Watson L. R. Sideris J. Bulluck J. Baranek G. T. (2018). Sensory features as predictors of adaptive behaviors: a comparative longitudinal study of children with autism spectrum disorder and other developmental disabilities. *Res. Dev. Disabil.* 81 103–112. 10.1016/j.ridd.2018.07.002 30060977 PMC7473611

[B136] Zhou H. Martinez E. Lin H. H. Yang R. Dale J. A. Liu K. Wang J. (2018). Inhibition of the prefrontal projection to the nucleus accumbens enhances pain sensitivity and affect. *Front. Cell. Neurosci.* 12:240. 10.3389/fncel.2018.00240 30150924 PMC6099095

[B137] Zhou R. Wang S. Zhu X. (2012). Prenatal ethanol exposure alters synaptic plasticity in the dorsolateral striatum of rat offspring via changing the reactivity of dopamine receptor. *PLoS One* 7:e42443. 10.1371/journal.pone.0042443 22916128 PMC3420902

